# Management of Medication-Related Osteonecrosis of the Jaw with Photobiomodulation and Minimal Surgical Intervention

**DOI:** 10.3390/dj11050127

**Published:** 2023-05-08

**Authors:** Marwan El Mobadder, Zuzanna Grzech-Lesniak, Wassim El Mobadder, Mohamad Rifai, Maher Ghandour, Samir Nammour

**Affiliations:** 1 Dental Surgery Department, Wroclaw Medical University, 50-425 Wroclaw, Poland; zuzanna.grzech-lesniak@student.umw.edu.pl; 2 Department of Dental Sciences, Faculty of Medicine, University of Liege, 4000 Liege, Belgium; 3 Department of Endodontics, Dental Specialist’ DS Polyclinics, Saida 1600, Lebanon; 4 Department of Periodontology, Faculty of Dental Medicine, Lebanese University, Beirut 6573/14, Lebanon; 5 Department of Orthopedics, Heidelberg University Hospital, 69120 Heidelberg, Germany

**Keywords:** osteonecrosis of the jaw, photobiomodulation, bone necrosis, laser, oral cavity, osteoporosis

## Abstract

Medication-related osteonecrosis of the jaw (MRONJ) is a relatively common pathology occurring in around 5% of patients taking bisphosphate and other antiresorptive or anti-angiogenic medications. Despite the efforts, as of today there is still no consensus on its management. In this case report, the successful management of stage II MRONJ was performed for an eighty-three-year-old female patient suffering from pain and alteration in her normal oral functions (swallowing and phonation). The treatment consisted of three sessions of photobiomodulation therapy (PBM), followed by minimal surgical intervention and three other sessions of PBM. PBM was applied on the sites of osteonecrosis with the follow parameters: 4 J/cm^2^; a power of 50 mW; 8 mm applicator diameter; a continuous contact mode. Irradiation was performed on three points, including the vestibular, occlusal and lingual parts of each of the bone exposure areas. Each point was irradiated for 40 s, and, in total, nine points were made per session, and nine sessions were conducted. To assess the pain, a visual analogue scale was used in which zero represented no pain at all and ten represented the greatest pain. At the first session and before any intervention, the patient stated that her pain was 8 out of 10. At the end of the treatment, a significant reduction in VAS was noted (2/10) and, clinically, a healing of the soft tissue in the previously exposed bone was observed. This case report suggests that the combination of PBM with surgical intervention is promising in the management of MRONJ.

## 1. Introduction

Medication-related osteonecrosis of the jaw (MRONJ) is a relatively common condition that occurs in around five percent of people taking bisphosphate and other antiresorptive anti-angiogenic medications, such as denosumab, bevacizumab, sunitinib and temsirolimus, for osteoporosis, cancer metastasis treatment or other conditions [1,2,3]. MRONJ is a progressive bone destruction occurring in the maxilla or the mandible that significantly affects the quality of life of patients and their overall health [4,5]. MRONJ presents several symptoms scaling from a mild sensation of pain to major swelling, suppuration and soft tissue ulceration, which can aggravate to severe pain, with an exposed necrotic bone able to threaten the patient’s life [5,6]. As for bisphosphonates and their implication in osteonecrosis, it is suggested that bisphosphonates act primarily by decreasing bone turnover via the inhibition of the osteoclast’s activity and inducing its apoptosis, thus leading to osteonecrosis of the jaw [7]. Moreover, it has been suggested that the presence of an infection in the site with this inhibited osteoclastic activity is what eventually results in bone necrosis [7].

As of today, the diagnosis of bisphosphonate-related and medication-related osteonecrosis of the jaw diagnosis is considered to be a major challenge for medical practitioners, among which dental surgeons play a primordial role [8]. It is agreed that MRONJ can be diagnosed by the presence of an exposed bone that did not heal after eight weeks, since this time frame is defined as the average time needed for soft tissue closure over the alveolar bone. Unfortunately, there is still no clear consensus nor agreed on guidelines for the management of MRONJ, and studies suggest that in a large number of cases MRONJ is unresponsive to medical treatment and surgical interventions [9]. Current treatment options include the administration of antibiotics [10], hyperbaric oxygen therapy (HBO) [11], bone resection [12], the use of low-power and high-power lasers [13], cell therapies [11,12,13], etc. Among these approaches, photobiomodulation (PBM) therapy seems to be promising in the management of MRONJ. PBM, previously known as low-level laser therapy, is defined as the therapeutic non-thermal use of light and/or an infrared light source to modulate living tissues. Despite its wide use, the mechanism of action of PBM is not fully understood; however, it is thought to involve the absorption of light by specific chromophores in the cell, such as cytochrome c oxidase, which leads to an increase in the production of cellular energy in the form of adenosine triphosphate (ATP) and improved cellular metabolism. Additionally, PBM therapy has been shown to modulate the expression of genes involved in inflammation and tissue repair and to stimulate the release of growth factors and other mediators of cellular healing. PBMT has been used to treat a wide range of conditions, including wound healing, pain and inflammation, and has been shown to be effective in both preclinical and clinical studies. However, more research is needed to fully understand the mechanisms of action and the optimal dosing for different conditions. PBMT might help to reduce the pain and inflammation associated with MRONJ and reduce the amount of pro-inflammatory cytokines and might also result in vasodilatation around the necrotic bone, which can help in the bone reparation process [14].

This case report describes the successful management of stage II medication-related osteonecrosis of the jaw. The treatment protocol consisted of both photobiomodulation therapy and minimal surgical intervention. To the best of our knowledge this case report presents a novel approach in the management of MRONJ.

## 2. Case Presentation

**Figure 1:**
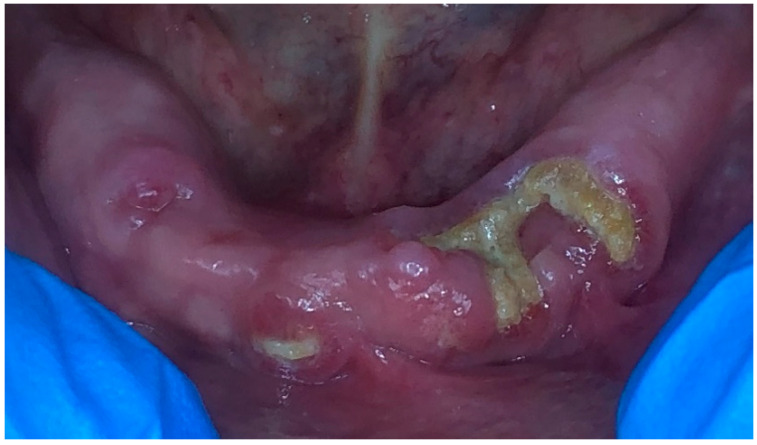
Clinical aspect of the mandibular arch at the first consultation and before treatment.

An 83-year-old female, non-smoking patient was referred to our clinic (M.E.M.) complaining of moderate to severe pain on the anterior region of the mandibular arch. Her medical history revealed that the patient suffered from osteoporosis and rheumatoid arthritis. The consultation for osteoporosis was made in the beginning of 2007, and the date of the first consultation in our office was on fifteen June 2012. The patient was taking bisphosphonate for a duration of 15 years. The bisphosphonate medication that the patient was taking was 150 mg of Ibandronate sodium (Adromux 150 mg tablets, Theramex Inc, India). According to the patient and her daughter, the chief complaint of the patient was moderate to severe pain on the anterior region of the mandible with an impairment of normal mastication leading to a decrease in her overall quality of life. A clinical examination revealed the presence of a localized exposed necrotic bone surrounded by an unhealed mucosa at different spots of the mandibular arch (Figure 1). The mucosa was painful upon percussion with a tendency of spontaneous bleeding around the exposed area. A retro-alveolar X-ray showed a spot of radio-opacity on the anterior region of the mandible. Based on the clinical and radiographical examination, stage II medication-related osteonecrosis of the jaw (MRONJ) was diagnosed based on the staging of the American Association of Oral and Maxillofacial Surgeons (AAOMS) (Table 1) [1]. A visual analogue scale (VAS) was used in order to assess the pain in which zero represents no pain at all and ten represents the greatest pain. The patient stated that the score of the VAS was 8/10 before any intervention. Prior to any intervention, a written informed consent was signed by the patient.

### 2.1. Treatment Protocol and Instructions

#### 2.1.1. Photobiomodulation Therapy

**Figure 2:**
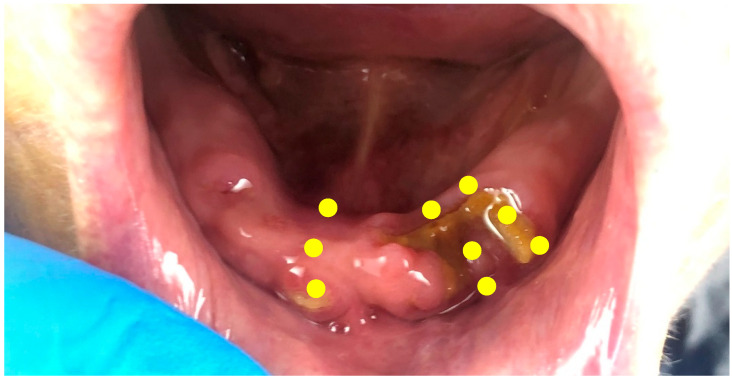
Clinical aspect of the mandibular arch immediately after the 3rd session of photobiomodulation therapy. The schematic yellow spots represent the areas of irradiation made in each of the nine sessions.

Three sessions of photobiomodulation (PBM) therapy were conducted, followed by a minimal local surgical intervention and three other sessions of PBM. The sessions of PBM therapy were conducted as follows: first, a sterile gauze imbibed with Povidone iodine (Betadine antiseptic solution, Mundipharma, Nicosia, Cyprus) was used in order to disinfect the entire mandibular arch with care on the exposed bone and the surrounding gingiva. Then, another sterile gauze was used to clean the povidone iodine to prepare the region for photobiomodulation therapy. At this stage, PBM was applied at the sites of the bone exposure with a 635 nm wavelength diode laser (Smart M, Lasotronix, Warsaw, Poland). The irradiation parameters on each of the points were as follows: 4 joules (J) per centimeter square (J/cm^2^); a power of 50 milliwatts (mW); an eight-millimeter (mm)-diameter applicator; irradiation for forty seconds (s) at each point; a continuous contact mode. Irradiation was made on three points, including the vestibular, occlusal and lingual parts, for each of the bone exposure areas. Therefore, in total, nine points were irradiated per session (Figure 2). The three sessions were performed in one week with a forty-eight-hour intermittent breaks between each session (for example, the sessions would be conducted on Monday, Wednesday and Friday). No antibiotics nor analgesics were prescribed for the patient. Moreover, the patient did not have any overdenture or prosthesis before, during or after treatment.

#### 2.1.2. Surgical Intervention

**Figure 3:**
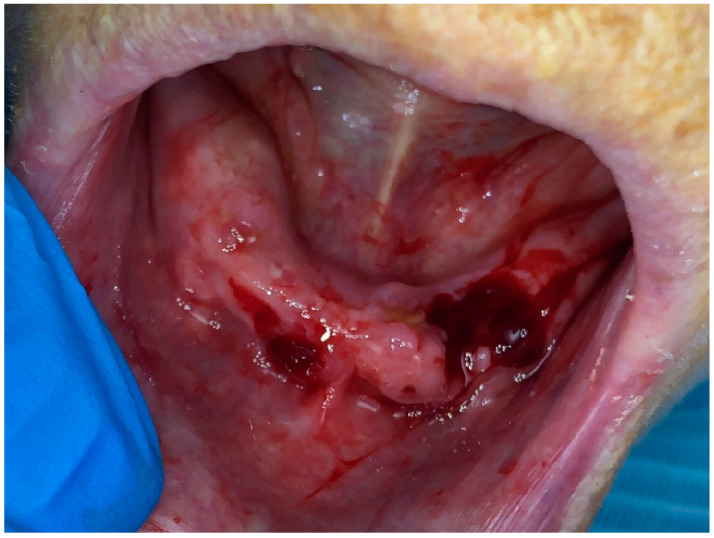
Clinical aspect of the mandibular arch immediately after the small surgical intervention.

After one week of the third session of photobiomodulation therapy, a minimal surgical intervention was performed at the exposure sites of the mandible. The surgical intervention was performed under local anesthesia and consisted of an excision of the necrotic bone using a sterile manual bone curette (HSI 100-4643 Bone Curette, Henry Schein Dental, Queens, New York City, NY, USA) followed by the use of a sterile round carbide burr mounted on a high-speed turbine. The excision was considered completed when bleeding from the bone started to appear, which suggests that a sound and living bone was reached. At this stage, a small incision of one millimeter was made on the gingival tissue surrounding the exposed bone in order to provoke bleeding. The blood was coagulated with a 980 nm diode laser (Smart M, Lasotronix, Warsaw, Poland) with the following parameters: a tip diameter of 200 μm (µm); a power of two watts, a continuous contact mode; an irradiation time of 20 s; a total energy density of 40 J/cm^2^. After treatment, the patient was asked not to rinse nor to brush this area for twenty-four hours. She was instructed to use chlorhexidine 0.12% mouthwash (Eludril Pro, Pierre Fabre, Castres, Midi-Pyrénées, France) after twenty-four hours of the intervention and asked to come in the next day for another session of photobiomodulation therapy (Figure 3). The same instructions were given to her daughter who was accompanying her in order to ensure the patient would follow the instructions at home. Moreover, the patient and her daughter were educated about the at-home management of MRONJ; the education was created based on the recommendations of the American Association of Oral and Maxillofacial Surgeons (AAOMS). It is also important to mention that no antibiotic medication was given to the patient and that no suturing was performed after treatment; rather, the blood coagulated and was left.

### 2.2. Result

**Figure 4:**
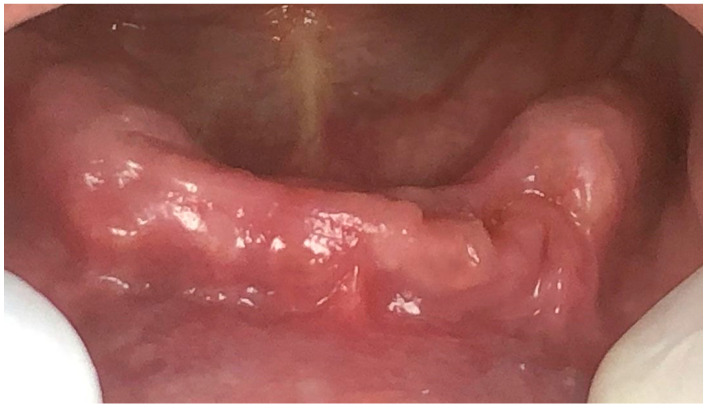
Clinical aspect of the mandibular arch 10 days following the intervention.

Follow-ups were conducted at twenty-four days and at three months following the last session of photobiomodulation therapy. At twenty-four days, the patient presented a healed mucosa with no signs of infection and a complete absence of any exposed bone (Figure 4). The patient scored 2/10 on the visual analogue scale and reported satisfaction with the overall results, with an absence of pain and a significant improvement in swallowing ability and overall quality of life. At three months post-operation, the patient scored two out of 10 again on the VAS and reported to be satisfied with an overall significant improvement in the quality of life. Moreover, the clinical aspect was similar of that seen at 24 days after surgery.

## 3. Discussion

In this case report, the suggested treatment protocol consisted of both nine sessions of PBM therapy and a minimal surgical intervention under local anesthesia. PBM therapy before and after the surgical intervention was performed in order to stimulate the re-vascularization of the tissues of the site, including the alveolar bone and the mucosa, to stimulate the wound healing and osteogenesis, and to attenuate and modulate the host response and, in particular, the inflammatory process. The possibility of stimulating re-vascularization with PBM has been confirmed in several studies. For instance, in an in vivo study by Ma et al. [16] on ischemic flaps in rats, it was demonstrated that PBM with near-infrared light enhances ischemic flap revascularization and increases flap viability compared to control groups. However, in their study [16], vascular endothelial growth factor staining showed that the difference in integrated optical density between the irradiated flaps with PBM therapy (low-level laser therapy) and the control flaps was not statistically significant, while α-smooth muscle actin and factor VIII staining showed significantly greater numbers of arterioles and capillaries in the irradiated flaps than in the control flaps [16]. PBM therapy was also used to attenuate the inflammatory process [17,18,19]. In this context, PBM therapy has been shown to have anti-inflammatory effects by reducing the production of pro-inflammatory molecules and increasing the production of anti-inflammatory molecules. These clinical effects of photobiomodulation therapy with an infrared light are well supported in the literature [17,18,19,20,21,22,23,24]. No antibiotics were prescribed in this case report. This confirms that the positive results were obtained from the treatment and not the antibiotherapy. PBMT appears to exert its anti-inflammatory effects via a variety of mechanisms, including reducing the production of pro-inflammatory molecules and increasing the production of anti-inflammatory molecules, modulating the activity of transcription factors, and increasing blood flow and angiogenesis [25]. As for the bone regeneration, studies have suggested that photobiomodulation therapy increases the proliferation and differentiation of osteoblasts and can also inhibit the activity of osteoclasts [25,26,27]. To reiterate, as photobiomodulation therapy increases blood flow and angiogenesis, the necessary transport of oxygen and nutrients to the healing tissue will occur, leading to better osteogenesis. Hence, in this case report, the use of photobiomodulation therapy before and after the minimal surgical intervention aimed to obtain this outcome.

As of today the exact mechanism of action of photobiomodulation therapy is not fully understood. However, the most accepted theory today is that photobiomodulation therapy, if used within proper parameters and protocols, results in an increase in the production of ATP in the mitochondria of the living cells due to the activation of cytochrome C oxidase (an enzyme in the cycle of ATP production), leading to an improved ability of cells to repair and regenerate. This increased energy production may also lead to a reduction in the production of pro-inflammatory molecules, such as cytokines and reactive oxygen species, and an increase in the production of anti-inflammatory molecules, such as interleukin-10. Marx et al. [28] introduced and described medication-related osteonecrosis of the jaw (MRONJ), and, since then, its treatment is still critically without standardized procedures [29,30]. Currently, the main strategies used to treat medication-related osteonecrosis of the jaw waver between non-surgical and surgical interventions [1,30]. The American Association of Oral and Maxillofacial Surgeons (AAMOS) stated that surgical interventions should only be recommended for stage III and above and that other options like oral rinses with chlorhexidine, improved dental hygiene and antibiotic therapy must be prioritized for stage I and II [1]. Interestingly, in this case report, a minimal surgical intervention was performed with additional PBM therapy, leading to the successful management of stage II MRONJ, a significant reduction in the sensation of pain and a significant improvement in the overall quality of life of the patient. In the aim of optimizing the management of MRONJ, one retrospective multicenter study evaluated different treatment strategies and factors affecting treatment outcomes. The study showed that several factors seem to play a major role in the success of interventions, such as the extent of bone resection achieved during the surgical intervention. Moreover, it was suggested that higher doses of an antiresorptive agent can negatively alter the effectiveness of the treatment and that the influence of discontinuing BP/Dmab during the treatment of MRONJ is still debated. Moreover, it was suggested that serum albumin level does not affect the outcome of treatment [31,32,33].

In addition to the studies mentioned earlier, two systematic reviews concluded that, although further evidence needs to be obtained, the laser-assisted management of MRONJ seems to be advantageous [34,35]. The use of laser irradiation in MRONJ can be divided into two groups: high-power laser irradiation for the surgical intervention of, mainly, the ablation of necrotic bone and low-power laser irradiation used for biostimulation effects [35].

Laser-assisted surgery is generally performed with the Er: YAG laser with a 2940 nm wavelength due to its strong affinity for hydroxyapatite and water, which leads to the effective ablation of bone with little to no increase in heating. In this context, studies have found that the rate of bone healing after Er:YAG laser irradiation can be similar or faster than the rate of bone healing after conventional bone cutting. This may be a result of the increased irregularity of the surface after laser irradiation, which makes it more conducive to the adhesion of blood components to bone tissue in the early stage of healing [36]. Moreover, it is critical to differentiate precisely between healthy bone and necrotic bone. Although the thorough removal of necrotic bone is essential, the excessive removal of healthy bone must be avoided to ensure that the jaw is not weakened. This facilitates dental or prosthetic rehabilitation [35]. As for PBMT, studies have demonstrated that low-level lasers present antibacterial potential and biostimulatory effects if applied to oral tissues within specific protocols and parameters [37]. Currently, photobiomodulation therapy has a wide range of applications, primarily in healing and the management of pain. Most studies on PBMT and its applications for bone use visible and infrared diode lasers such as Nd: YAG lasers and diode lasers. In this context, an international multidisciplinary panel of clinicians and researchers with expertise in the areas of supportive care in cancer and/or PBM clinical application and dosimetry has suggested PBM protocols and parameters to be used in osteonecrosis of the jaw [38,39]. Based on their recommendations, a continued treatment at least two or three times a week until osteonecrosis symptoms improve must be conducted, and daily treatment is recommended in combination with other medical/surgical treatment approaches if necessary [39].

One possible limitation of this study is the absence of a pre-operative and post-operative radiographic examination such as a peri-apical X-ray, panoramic X-ray or a CBCT. Hence, this case report shows that the combination of both surgical intervention and PBM before and after the surgical intervention results in a significant improvement in the overall healing of stage II medication-related osteonecrosis of the jaw without the need for an aggressive surgical intervention. For this reason, we invite further studies to be performed with our suggested treatment protocol and parameters.

## 4. Conclusions

Within the limitations of this case report, PBMT as an additional approach to a minimal surgical intervention in stage II medication-related osteonecrosis of the jaw can be considered an effective therapeutic approach if used within our suggested irradiation parameters and treatment protocol. However, further studies are required to confirm the findings of this case report.

## Figures and Tables

**Table 1:** Classification of medication-related osteonecrosis of the jaw based on the American Association of Oral and Maxillofacial Surgeons position paper on the disease from 2014 [15].

Stage	Description	Treatment
At risk	Asymptomatic patients with no apparent necrotic bone who have been treated with IV or oral antiresorptive or antiangiogenic drugs	No treatment indicated Patient education See dentist regularly
Stage 0	Non-specific symptoms or clinical/radiographic findings suggesting bone necrosis No evidence of exposed necrotic bone, but sinus tract or fistula may be present	Antibacterial mouth wash Pain medication and antibiotics if symptomatic Patient education
Stage 1	No evidence of pain/infection Exposed necrotic bone	Antibacterial mouth wash Pain medication and antibiotics Debridement Patient education
Stage 2	Pain and/or infection Exposed and necrotic bone or a fistula that probes to bone	Antibacterial mouth wash Pain medication and antibiotics Debridement Patient education
Stage 3	Exposed and necrotic bone or fistulas that probe to bone with evidence of infection and at least 1 of the following: Exposed necrotic bone extending beyond the region of alveolar bone Pathologic fracture Extraoral fistula Oral–antral or oral–nasal communication Osteolysis extending to the inferior border of the mandible or sinus floor	Antibacterial mouth wash Pain medication and antibiotics Debridement Patient education

## Data Availability

Data are available upon reasonable request from the corresponding author (M.E.M).
